# 

**Published:** 1895-09-21

**Authors:** 


**Notices of Births, Deaths, and Marriages** are inserted in this column at a charge of 2s. 6d. for an announcement not exceeding four lines.

**Notice to Advertisers and Subscribers.**

All Advertisements, Orders for Copies of The Hospital and Business Communications should be addressed to **The Manager, NOT TO THE EDITOR,** The Hospital, 428, Strand, London, W.C.

The Cost of Subscription, post free, is as follows:—Per week, 2½d.; per quarter, 2s. 8d.; per half-year, 5s. 3d.; per annum, 10s. 6d. Foreign:—Per Annum, 15s. 2d.; payable, in all cases, in advance.

### NOTICE TO CORRESPONDENTS.

All MS., Letters, Books for Review, and other matters intended for the Editor should be addressed The Editor, The Lodge, Porchester Square, London, W.

The Editor cannot undertake to return rejected MS., even when accompanied by stamped directed envelope.

**"Burdett's Hospital Annual," 1895.**

Sixth year of publication. 950 pp. Scarlet cloth, gilt lettered. Price 5s. A most complete guide to British, Colonial, Indian, and American Hospitals, Dispensaries, Nursing and Convalescent Institutions, and Asylums. A few copies of the "Annual" for 1894 may still be obtained on application to the Publishers, The Scientific Press (Limited), 428, Strand, London, W.C.

**NOTICE.—Vol. XVII. of "The Hospital" (October, 1894, to April, 1895), in handsome cloth binding, is on sale at the Office of this Journal. Price 6s. Cases for binding the Numbers contained in this Volume, 1s. 6d. Index to Vol. XVII., 2½d., post free.**

**The Monthly Part for September is now ready, price 6d.; by post, 8d.**

### SCRAPS AND GLEANINGS.

THE total amount collected this year by the friendly societies' hospital movement at Barnstaple amounted to £152.

THE 21st annual report of the Hull Hospital Sunday Fund points out that, taking into consideration the open state of trade, it was a cause for thankfulness that the fund had reached the amount of £758, and that it had not further declined was a proof that Hospital Sunday maintained its popularity as a convenient and successful means of adding to the funds of the Hull medical charities.

THE Local Government Board's sanction is to be asked by the Tees Port Sanitary Authority to use the Tees Floating Hospital for the reception of cholera cases arriving from the sea.

THE Terrol Company have gained a gold medal for their product (Terrol) at the World's Exhibition, Amsterdam.

THE Church of England Incorporated Society for Providing Homes for Waifs and Strays has received the offer of £500 to establish in Lincoln a home for boys and girls for that diocese.

A CONVALESCENT home is to be founded for girls discharged from hospitals at Garches, where an estate has been bequeathed for this purpose by Madame Davaine.

THE Council of the Hospital Sunday Fund issued a special appeal to the various metropolitan contributing workshops and business houses urging the necessity for increased effort, with a view to obtaining an ultimate total of contributions not falling short of those received last year, which amounted to £20,000.

UNDER the patronage of Lady Magdalen Williams Bulkeley a grand charity calico ball was held this month at the Town Hall, Beaumaris, in aid of the Beaumaris District Nursing Institution.

THE receipts of the hospital Saturday and Sunday collection in aid of the Victoria Hospital, Folkestone, brought in over £60 to that deserving institution. The amount fell short by about £30 of that collected last year.

THE debt balance at the bank for last quarter at the Cumberland Infirmary amounted to £1,500. But at the present time it stands at £500.

ON the occasion of the forthcoming opening of the Southport New Infirmary it is estimated that the festivities will cost over £1,000.

THE 19th annual report of the Stockton Hospital Committee is of a pleasing and satisfactory nature. The hospital has done much good work during the period under review, and the finances for the year show over £700 to the good. The laundry, which is now completed, has been erected for nearly £40 under the original estimate.

THE private nursing scheme in connection with the Cumberland Infirmary has brought in a net profit of £55 to the funds.

### THE STUDENT OF MEDICINE.

### APPOINTMENTS.

W. M. Clendinnen, M.R.C.S.Eng., L.R.C.P.Lond., appointed Medical Officer of Health to the Coseley District Council. A. D. Crawford, M.B., C.M.Glasg., appointed Medical Officer for the Marston Green Cottage House of the Parish of Birmingham. E. I. Day, L.R.C.P., appointed Medical Officer and Public Vaccinator for the Fourth District, Risbridge Union. Emily W. Dickson, M.B., B.Ch., appointed Assistant to the Master of the Coombe Lying-in Hospital, Dublin. C. A. Etches, L.R.C.P.Edin., L.R.C.S. Edin., appointed Medical Officer for the Helperby District of the Easingwold Union. Dr. J. P. W. Freeman, appointed Medical Officer for the Fifth District of the Andover Union. G. C. Hall, L.S.A., M.R.C.S.Eng., appointed Medical Officer for the Ambersley District of the Droitwich Union. S. H. Hall, M.B., C.M.Edin., appointed Medical Officer of the Fusehill and Harraby Hill Workhouses of the Carlisle Union. R. J. Harvey, L.R.C.P.I., L.R.C.S.I., appointed Assistant Surgeon to the Richmond Hospital, Dublin. J. H. Head, B.A., M.D., B.Ch., B.A.O.Dubl., appointed House Surgeon to the Morpeth Dispensary. G. Hill, L.R.C.P.Edin., L.R.C.S. Edin., appointed Medical Officer of Health to the Holme Cultram Urban District Council. E. O. Jago, M.R.C.S.Eng., L.S.A.Lond., appointed Medical Officer of Health to the Iream Urban District Council. T. J. P. Jenkins, M.R.C.S.Eng., L.R.C.P.Lond., appointed Medical Officer for the South-Eastern District of the Freebridge Lynn Union. G. J. Johnston, M.A., M.B., B.Ch., B.A.O., appointed Assistant Surgeon to Richmond Hospital, Dublin. W. G. Laws, F.R.C.S. Eng., appointed Honorary Surgeon to the Nottingham and Midland Eye Infirmary. J. A. Macdonald, M.B., C.M.Glasg., appointed Medical Officer for the Denton District of the Grantham Out-relief Union. E. C. Osborn, L.R.C.P.Lond., appointed Assistant Medical Officer to the Kensington Workhouse and Infirmary. A. H. D. Salt, L.R.C.P.Edin., L.R.C.S.Edin., appointed Medical Officer for the Sturry District of the Blear Union. A. L. Saunders, M.R.C.S.Eng., L.R.C.P.Lond., appointed Medical Officer of the Workhouse and the Eastern District of the Freebridge Lynn Union. H. Shaw, B.A., M.B.Camb., D.P.H., appointed Medical Superintendent of the County Asylum of the Isle of Wight. R. C. J. Stevens, M.R.C.S.Eng., L.R.C.P. Lond., appointed Assistant House Surgeon to the Devon and Exeter Hospital. F. W. Style, M.R.C.S.Eng., L.R.C.P.Lond., appointed Medical Officer for the South Brent District of the Totnes Union. Dr. J. Taylor, appointed Medical Officer of Health to the Chester-le-Street Rural District Council. T. W. Thomas, L.S.A.Lond., M.R.C.S.Eng., appointed Medical Officer and Public Vaccinator for the Caerphilly Urban District of the Pontypridd Union. Dr. Thurison, appointed District Medical Officer to the St. George's Union. R. J. Hillier has been appointed Senior House Surgeon to the West Ham Hospital. A. P. Woollright, L.S.A.Lond., has been appointed Junior House Surgeon to the West Ham Hospital.

### VACANCIES.

The following vacancies are announced this week, the amount of salary in each case being given after the name of the institution:—

### Resident Medical Officers.

Belgrave Hospital for Children, 77 and 79, Gloucester Street, S.W.—House Surgeon. Board, fuel, and lights provided. Applications, endorsed "House Surgeon," to the Honorary Secretary at the Hospital by September 28th.

Birmingham and Midland Eye Hospital.—Assistant House Surgeon. Salary £50 per annum, with apartments and board. Applications to the Chairman of the Medical Board by September 30th.

Carmarthenshire Infirmary.—Resident Medical Officer, unmarried. Salary £100, with board, lodging, and washing. Knowledge of Welsh desirable. Applications to B. Spivey, Secretary, Barns Row, Carmarthen, by September 24th.

Clayton Hospital and Wakefield General Dispensary.—Junior House Surgeon, registered and unmarried. Honorarium £40 per annum, with board, lodging, and washing. Applications, with not more than three recent testimonials, to the Honorary Secretary, Clayton Hospital, Wakefield, by September 26th.

Durham County Asylum.—Junior Medical Officer. Salary £100 per annum, with board and lodging. Applications, with not more than three recent testimonials, to the Superintendent, Durham County Asylum, Winterton, Ferryhill, by September 28th.

Essex and Colchester Hospital.—House Surgeon, doubly qualified. Salary £80 per annum, with board and lodging in the hospital. Applications to the Committee by October 18th.

General Hospital, Birmingham.—Two Assistant House Surgeons, must possess a surgical qualification. Appointment for six months. No salary, but residence, board, and washing provided. Applications to Howard J. Collins, House Governor, by September 28th.

Jaffray Suburban Branch of the General Hospital, Gravelly Hill, near Birmingham.—Resident Medical Surgical Officer, doubly qualified. Salary £150 per annum, with board, residence, and washing. Applications to Howard J. Collins, House Governor, General Hospital, Birmingham, by September 28th.

Queen Charlotte's Lying-in Hospital, Marylebone Road, N.W.—Resident Medical Officer. Appointment for four months. Salary at the rate of £60 per annum, with board and residence. Applications to the Secretary by September 23rd.

West London Hospital, Hammersmith Road, W.—House Surgeon. Appointment for six months. Board and lodging provided. Applications to R. J. Gilbert, Secretary Superintendent, by September 25th.

### Non-Resident Medical Officers.

Carnarvon Joint Sanitary District.—Medical Officer of Health, must be between 25 and 40 years of age, doubly qualified. Must devote his whole time to the duties, and have a knowledge of the Welsh language. Appointment for five years. Salary £694 per annum, inclusive of all expenses, except those incurred for such books, stationery, and apparatus required in the performance of the duties. Applications, endorsed "Application for Office of M. O. Health," to J. H. Thomas, Clerk to the Joint Committee, 14, Market Street, Carnarvon, by October 16th.

County of Brecon.—County Analyst. Retaining fee £10 10s., and a fee of 10s. 6d. for the analysis of every sample. Applications to H. Edgar Thomas, Clerk to the County Council, County Hall, Brecon, by October 2nd.

St. Bartholomew's Hospital and College.—Assistant Demonstrator of Chemistry. Applications to Thomas W. Shore, Warden, before September 21st.

